# Prognostic significance of subsequent decline in LVEF in heart failure with improved ejection fraction − A report from the CHART-2 study −

**DOI:** 10.1016/j.ijcha.2026.101877

**Published:** 2026-01-23

**Authors:** Takuya Takigahira, Kotaro Nochioka, Satoshi Miyata, Takashi Shiroto, Takumi Inoue, Kai Susukita, Hideka Hayashi, Hiroyuki Takahama, Jun Takahashi, Hiroaki Shimokawa, Satoshi Yasuda

**Affiliations:** a Teikyo University Graduate School of Public Health, Japan; b Department of Cardiovascular Medicine, Tohoku University Graduate School of Medicine, Japan; c International University of Health and Welfare, Japan

**Keywords:** Heart failure, Prognosis, Left ventricular ejection fraction, Epidemiology

## Abstract

**Background:**

Some patients of heart failure with improved ejection fraction (HFimpEF) have subsequent decline in left ventricular ejection fraction (LVEF) after improvement, and their prognosis is uncertain.

**Aims:**

We aimed to examine the clinical characteristics and long-term prognosis of this sub-population of HFimpEF.

**Methods:**

We examined 399 consecutive patients with HF with reduced ejection fraction (HFrEF, LVEF ≤ 40 %) with LVEF data at both baseline and follow-up in the CHART-2 Study. We classified them as follows; persistent HFrEF group (LVEF ≤ 40 % at 1-year and 2-year follow-up, n = 238), temporary HFimpEF group (≥10 % increase from baseline with LVEF > 40 % at 1-year follow-up but LVEF ≤ 40 % at 2-year follow-up, n = 22), and persistent HFimpEF group (≥10 % increase from baseline with LVEF > 40 % at 1-year follow-up, and LVEF > 40 % at 2-year follow-up, n = 139).

**Results:**

The temporary HFimpEF group (adjusted hazard ratio: 2.95; 95 % CI: 1.55–5.63) and the persistent HFrEF group (2.53; 1.75–3.67) were associated with increased risks for the composite of cardiovascular death and HF hospitalization. The risk factors for decline in LVEF included LVEF (adjusted odds ratio: 0.80; 95 %CI: 0.69–0.90), LV end-diastolic dimension (LVDd) (1.14; 1.05–1.25), B-type natriuretic peptide (BNP) levels (1.04 per 10 pg/mL increase; 1.00–1.08), estimated glomerular filtration rate (eGFR) levels (0.95; 0.92–0.99) and serum sodium levels (0.70; 0.50–0.91) at 1-year follow-up.

**Conclusions:**

These results indicate that patients with HFrecEF account for 23% of those with HFrEF and that 12% of them have subsequent decline in LVEF associated with similar worse prognosis as in those with persistent HFrEF.

## 1 Introduction

Heart failure (HF) is a global health burden affecting about 26 million people worldwide [1]. In clinical practice, measuring left ventricular ejection fraction (LVEF) is essential for selecting treatment and predicting prognosis in patients with HF [2].

The professional guidelines recommend to clinicians to categorize HF into 3 categories based on LVEF, including HF with reduced ejection fraction　(HFrEF), HF with mildly reduced ejection fraction (HFmrEF), and HF with preserved ejection fraction (HFpEF) [3]. However, based on observations of LVEF improvement in clinical trials by medications,[4], [5] heart failure with improved ejection fraction (HFimpEF) was proposed as a new category which has distinct characteristics with better prognosis from other 3 categories as defined as follows; 1) a baseline LVEF ≤ 40 %, 2) a ≥ 10 % increase from baseline LVEF, and 3) second measurement of LVEF > 40 % [6], [7], [8], [9]. In contract, previous studies demonstrated that even though the improvement of LVEF is observed in patients in HFimpEF, several biomarkers, such as B-type natriuretic peptide (BNP) and soluble fms-like tyrosine kinase receptor-1(sFlt-1), remain abnormal, indicating that LVEF improvement alone does not necessarily indicate cardiac normalization [6], [10], [11], [12]. Indeed, subsequent decline in LVEF is observed in some patients with HFimpEF although clinical characteristics and long-term prognosis of this sub-population remain to be elucidated.

In the present exploratory study, we thus aimed to examine the clinical characteristics and long-term prognosis of patients with HFimpEF with subsequent decline in LVEF in our multicenter observational study, the Chronic Heart Failure Registry and Analysis-2 (CHART-2) study [13].

## 2 Methods

### 2.1 Study setting

**Fig. 1:**
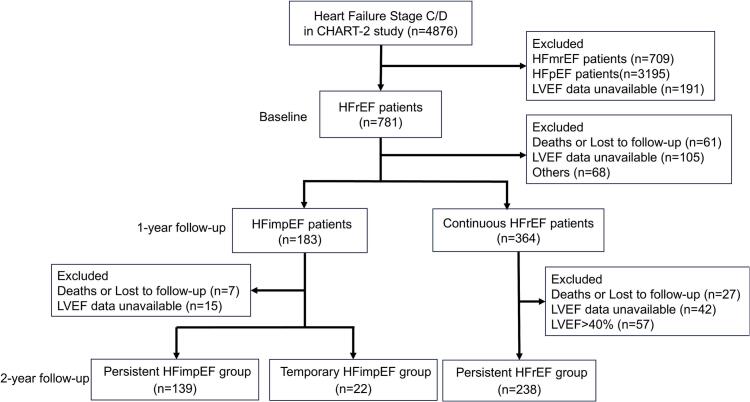
**Flowchart of patient selection and classification** Abbreviations: HFimpEF, heart failure with improved ejection fraction; HFmrEF, heart failure with mildy-reduced ejection fraction; HFpEF, heart failure with preserved ejection fraction; HFrEF, heart failure with reduced ejection fraction; LVEF, left ventricular ejection fraction.

This is a post hoc analysis of the CHART-2 study. The CHART-2 study is a prospective cohort study of chronic HF in Japan. The details of the study were described previously [13]. Briefly, a total of 10,209 consecutive patients aged ≥ 20 years with chronic HF were enrolled from 24 hospitals between October 2006 and March 2010. According to the ACC/AHA guidelines, patients were classified as Stage A/B heart failure (n = 5,333) and Stage C/D heart failure (n = 4,876) [13]. The diagnosis of HF was made by experienced cardiologists based on the Framingham criteria [14]. Ischemic heart disease was defined as the presence of significant coronary artery disease, characterized by flow-limited stenosis requiring revascularization. The study protocol was approved by the regional ethics committees of each participating hospital, and written informed consent was obtained from all patients. Fig. 1 shows the study diagram. Of 4,876 patients with Stage C/D in the CHART-2 study, we excluded 3,195 patients with HFpEF (LVEF ≥ 50 %), 709 patients with HFmrEF (LVEF41-49 %) and 191 had missing echocardiographic data at enrollment. A total of 781 patients with HFrEF (LVEF ≤ 40 %) at baseline were included. At the 1-year follow-up, 364 patients remained classified as continuous HFrEF (LVEF ≤ 40 %), while 183 met the criteria for HFimpEF (LVEF > 40 % with a ≥ 10 % increase from baseline). Among the continuous HFrEF group, 27 patients died or lost to follow-up, 42 had missing echocardiographic data, and 57 were reclassified into other groups (LVEF > 40 %). At the 2-year follow-up, 238 patients were categorized as having persistent HFrEF (LVEF ≤ 40 %). Of the 183 patients with HFimpEF at 1-year, 7 died or lost to follow-up, 15 had missing echocardiographic data, 22 were reclassified as temporary HFimpEF (LVEF ≤ 40 %), and 139 were categorized as persistent HFimpEF (LVEF > 40 %). The final study population included 399 patients: 238 with persistent HFrEF, 22 with temporary HFimpEF, and 139 with persistent HFimpEF. The primary outcome was the composite of cardiovascular death and HF hospitalization.

### 2.2 Data collection and follow-up

Patient information, including demographics, medical history, clinical examinations, echocardiography, and angiography, was recorded at the time of enrollment. Follow-up was made at least once a year by clinical research coordinators by means of review of medical records, surveys and telephone interviews.

### 2.3 Measurement of LVEF

We measured LVEF at baseline and annually at each hospital in clinical setting followed by the European Society of Echocardiology guidelines [15]. Core laboratory measurement of LVEF was not conducted. Of the 399 patients with HFrEF, we classified them as follows; persistent HFrEF group (LVEF ≤ 40 % at 1-year and 2-year follow-up, n = 238), temporary HFimpEF group (≥10 % increase from baseline with LVEF > 40 % at 1-year follow-up but LVEF ≤ 40 % at 2-year follow-up, n = 22), and persistent HFimpEF group (≥10 % increase from baseline with LVEF > 40 % at both 1- and 2-year follow-up, n = 139) (Fig. 1).

### 2.4 Statistical analysis

Continuous variables were presented as mean with standard deviation or median with interquartile range (IQR) and categorical variables were expressed as number with percentage as appropriate. Comparisons among groups were performed using the Kruskal-Wallis rank sum test or Analysis of Variance (ANOVA) for continuous variables, and Fisher’s exact test for categorical variables as appropriate.

Event-free survival curves were described using the Kaplan-Mier method with log-rank test. In the survival analysis, patients were censored if they did not experience cardiovascular death or HF hospitalization by the end of the study period. Cox regression analyses were used to calculate the estimated hazard ratio (HR) with a 95 % confidence interval (CI). We built the following three models; model 1, unadjusted; model 2, adjusted for age and sex; and model 3, adjusted for age, sex, BNP levels, serum sodium, hemoglobin, blood urea nitrogen (BUN), history of ischemic heart disease, cancer, atrial fibrillation, and New York Heart Association (NYHA) class Ⅲ-Ⅳ at 1-year follow-up based on literature search [16], [17]. The proportional hazards assumption was verified using Schoenfeld residuals test and log–log survival plot.

To examine the risk factors for decline in LVEF, the following candidate variables were selected; age, female sex, heart rate, systolic blood pressure (BP), diastolic BP, NYHA class Ⅲ-Ⅳ, history of ischemic heart disease, atrial fibrillation, cancer, anemia and diabetes mellitus, LVEF, LV end-diastolic dimension (LVDd), interventricular septal thickness at end-diastole (IVSTD), posterior wall thickness at end-diastole (PWD), left atrial diameter (LAD), LV mass (LVM), BNP levels, estimated glomerular filtration rate (eGFR), serum sodium, serum potassium at the 1-year follow-up. Univariable logistic regression analysis was first conducted for each of the candidate variables to identify individual associations with the decline in LVEF. Subsequently, the multivariable model was determined based on Akaike’s Information Criterion (AIC) to ensure an optimal balance between model fit and complexity. After selecting the multivariable model, we performed internal validation to assess model stability and potential overfitting. Model discrimination was evaluated using the area under the receiver operating characteristic curve (AUC). Internal validation was conducted using two resampling approaches: 10‐fold cross‐validation and bootstrap resampling (1,000 iterations). All tests were two-sided, and the significance level was set at p < 0.05. Statistical analyses were performed using R, version 4.5.2.

## 3 Results

### 3.1 Clinical characteristics

At 1-year follow-up, improvement of LVEF was observed in 183 patients (23 %) among the 781 patients with HFrEF patients at baseline. We identified 183 patients with HFimpEF at 1-year follow-up, and among them, a decline in LVEF was observed in 22 patients (12 %) at 2-year follow-up (Fig. 1). Table 1 shows clinical characteristics at 1-year follow-up of the 399 patients stratified by LVEF at 2-year follow-up; persistent HFimpEF group (n = 139), temporary HFimpEF group (n = 22), and persistent HFrEF group (n = 238). Significant changes in LVEF were observed during the initial 2 years, whereas the LVEF remained clinically stable in the three groups (Supplemental Fig. 1). The temporary HFimpEF group had less women, lower systolic BP, LVEF, and eGFR. The temporary HFimpEF group also had higher proportion of ischemic heart disease, higher LVDd, LVDs, LVM, and BUN. The persistent HFimpEF group was treated with less beta-blockers, renin-angiotensin-system inhibitors, and diuretics than the temporary HFimpEF and the persistent HFrEF groups (Table1**)**. There were no significant changes in the use of renin-angiotensin system inhibitors and beta-blockers from baseline to 2-year follow-up in all three groups (Supplemental Table 1). Additionally, no significant differences were observed among the groups regarding the proportion of patients with valvular surgery or the distribution of cardiomyopathy subtypes (Supplemental Table 2 & 3).

**Table 1:** Clinical characteristics at 1-year follow-up of the three groups.

**Characteristic**	**Persistent HFimpEF group (N = 139)**	**Temporary HFimpEF group(N = 22)**	**Persistent HFrEF group(N = 238)**	**p-value**
Age (years)	66 ± 13	68 ± 11	66 ± 12	0.660
Female sex, n (%)	43 (30.9 %)	3 (13.6 %)	45 (18.9 %)	0.019
BMI (kg/m^2^)	23.1 ± 3.3	23.4 ± 3.4	23.6 ± 4.1	0.553
Heart Rate (bpm)	71 ± 14	70 ± 11	73 ± 14	0.393
Systolic BP (mmHg)	125 ± 18	116 ± 18	113 ± 17	<0.001
Diastolic BP (mmHg)	71 ± 12	69 ± 10	68 ± 10	0.031
NYHA class, n (%)				0.005
Ⅰ	28 (23.0 %)	2 (10.5 %)	19 (9.4 %)	
Ⅱ	85 (69.7 %)	12 (63.2 %)	155 (76.4 %)	
Ⅲ	8 (6.6 %)	5 (26.3 %)	26 (12.8 %)	
Ⅳ	1 (0.8 %)	0 (0.0 %)	3 (1.5 %)	
**HF etiology**				
Ischemic heart disease, n (%)	51 (36.7 %)	11 (50.0 %)	130 (54.6 %)	0.003
Valvular heart disease, n (%)	37 (26.6 %)	4 (18.2 %)	44 (18.5 %)	0.171
Cardiomyopathy, n (%)	55 (39.6 %)	9 (40.9 %)	107 (45.0 %)	0.617
**Clinical history**				
Hypertension, n (%)	126 (90.6 %)	19 (86.4 %)	206 (86.6 %)	0.446
Atrial fibrillation, n (%)	54 (38.8 %)	10 (45.5 %)	88 (37.0 %)	0.698
Diabetes mellitus, n (%)	63 (45.3 %)	9 (40.9 %)	112 (47.1 %)	0.837
Cancer, n (%)	16 (11.5 %)	3 (13.6 %)	22 (9.2 %)	0.567
PMI, n (%)	5 (3.6 %)	3 (13.6 %)	26 (10.9 %)	0.019
ICD/CRTD, n (%)	6 (4.3 %)	2 (9.1 %)	54 (22.7 %)	<0.001
**Echocardiography**				
LVEF (%)	55 ± 8	48 ± 7	29 ± 6	
LVDd (mm)	53 ± 8	60 ± 8	65 ± 10	<0.001
LVDs (mm)	37 ± 7	46 ± 7	56 ± 10	<0.001
IVSTD (mm)	10.86 ± 2.13	10.10 ± 2.18	9.26 ± 2.69	<0.001
PWD (mm)	10.51 ± 1.91	9.96 ± 2.30	9.54 ± 2.17	<0.001
LAD (mm)	42 ± 8	44 ± 12	45 ± 9	0.014
LVM (g)	221 ± 79	257 ± 106	267 ± 96	<0.001
**Laboratory**				
BNP (pg/mL)	78 (31, 174)	82 (52, 229)	189 (96, 394)	<0.001
Serum sodium (mEq/L)	141 ± 2	140 ± 3	141 ± 3	0.125
Serum potassium (mEq/L)	4.34 ± 0.49	4.41 ± 0.27	4.41 ± 0.42	0.398
LDL-C (mg/dL)	105 ± 29	94 ± 24	104 ± 28	0.161
Albumin (g/dL)	4.19 ± 0.44	3.98 ± 0.43	4.18 ± 0.38	0.129
Hemoglobin (g/dL)	13.28 ± 1.67	13.24 ± 2.28	13.60 ± 2.04	0.266
eGFR (mL/min/1.73 m^2^)	63 ± 22	56 ± 16	57 ± 21	0.030
BUN (mg/dL)	19 ± 9	21 ± 6	22 ± 11	0.025
**Medication**				
Bata-blockers, n (%)	92 (66.2 %)	18 (81.8 %)	189 (79.4 %)	0.014
RAS inhibitors, n (%)	107 (77.0 %)	21 (95.5 %)	202 (84.9 %)	0.043
Diuretics, n (%)	98 (70.5 %)	19 (86.4 %)	200 (84.0 %)	0.006
CCBs, n (%)	38 (27.3 %)	1 (4.5 %)	32 (13.4 %)	<0.001
Digitalis, n (%)	36 (25.9 %)	8 (36.4 %)	69 (29.0 %)	0.520

Continuous variables are expressed as mean with standard deviation, expect BNP levels, which are expressed as median with interquartile range.

Abbreviations: BMI, body mass index; BNP, B-type natriuretic peptide; BP, blood pressure; BUN, blood urea nitrogen; CCB, calcium channel blockers; CRTD, cardiac resynchronization therapy defibrillator; eGFR, estimated glomerular filtration rate; HFimpEF, heart failure with improved ejection fraction; HFrEF, heart failure with reduced ejection fraction; ICD, implantable cardioverter defibrillator; IVSTD, interventricular septal thickness at end-diastole; LAD, left atrial diameter; LDL-C, low-density lipoprotein cholesterol; LVDd, left ventricular diastolic dimension; LVDs, left ventricular systolic dimension; LVEF, left ventricular ejection fraction; LVM, left ventricular mass; NYHA, New York Heart Association; PMI, pacemaker implantation; PWD, posterior wall diameter; RAS, renin-angiotensin system.

### 3.2 Long-term prognosis of HFimpEF

**Fig. 2:**
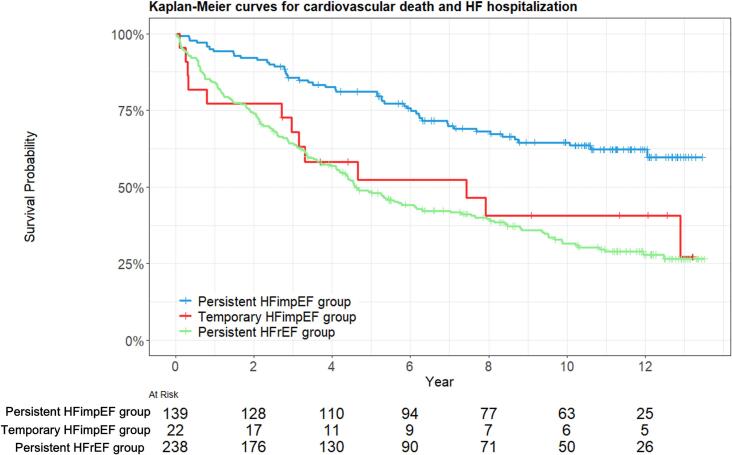
**Kaplan-Meier survival curves for cardiovascular death and heart failure (HF) hospitalization** The Kaplan-Meier curves depict survival probabilities over time (years) among the 3 groups; persistent HFimpEF (blue), temporary HFimpEF (red), and persistent HFrEF (green) groups. Abbreviations: See Fig. 1. (For interpretation of the references to colour in this figure legend, the reader is referred to the web version of this article.)

During the median follow-up period of 5.69 years (IQR:2.60–10.64 years), 220 of 399 patients (55 %) had composite outcomes of cardiovascular death and HF hospitalization. Fig. 2 shows the Kaplan-Meier curve estimates for cardiovascular death and HF hospitalization in the 3 groups. The persistent HFimpEF group showed a lowest incidence of composite outcome (/1,000 person-years) (persistent HFimpEF group 43.9, temporary HFimpEF group 100.7, and persistent HFrEF group 121.9; log-rank P < 0.001). Particularly, the temporary HFimpEF group had a higher incidence of hospitalization for HF during the improvement period of LVEF. Furthermore, after adjusting for various potential confounders, compared with the persistent HFimpEF group, both the temporary HFimpEF and the persistent HFrEF groups were associated with an increased risk of the composite outcome across the 3 Cox models (adjusted HR for temporary HFimpEF, 2.95, 95 % CI: 1.55–5.63, P = 0.001; adjusted HR for persistent HFrEF, 2.53, 95 % CI: 1.75–3.67, P < 0.001) (Table2).

**Table 2:** Cox regression model analysis for composite of cardiovascular death and HF hospitalization.

	Number of events	**Model 1**			**Model 2**			**Model 3**		
		HR	95 % CI	p-value	aHR	95 % CI	p-value	aHR	95 % CI	p-value
Persistent HFimpEF group	49/139	reference			reference			reference		
Temporary HFimpEF group	13/22	2.25	1.22–4.15	0.010	2.20	1.19–4.08	0.012	2.95	1.55–5.63	0.001
Persistent HFrEF group	158/238	2.64	1.91–3.64	<0.001	2.63	1.90–3.64	<0.001	2.53	1.75–3.67	<0.001

Model 1: non-adjusted model. Model 2: Adjusted for age and sex. Model 3: Adjusted for age, sex, BNP levels, serum sodium, hemoglobin, blood urea nitrogen (BUN), history of ischemic heart disease, cancer, atrial fibrillation, and NYHA class Ⅲ-Ⅳ.

Abbreviations: 95%CI, 95% confidence interval; aHR, adjusted hazard ratio; HFimpEF, heart failure with improved ejection fraction; HFrEF, heart failure with reduced ejection fraction

### 3.3 Risk factors for decline in LVEF in HFimpEF

A total of 22 events of decline in LVEF were observed at the 2-year follow-up. Systolic BP (odds ratio (OR), 0.97; 95 %CI: 0.94–1.00, P = 0.038), NYHA class Ⅲ-Ⅳ (4.48; 1.24–15.03, P = 0.016), LVEF (0.85; 0.78–0.92, P < 0.001), LVDd (1.11; 1.05–1.19, P < 0.001) and BNP (1.03 per 10 pg/mL increase; 1.00–1.05, P = 0.028) were associated with decline in LVEF in the univariable model. In multivariable model with AIC selection, LVEF (adjusted OR, 0.80; 95 %CI: 0.69–0.90, P < 0.001), LVDd (1.14; 1.05–1.25, P = 0.003), BNP (1.04 per 10 pg/mL increase; 1.00–1.08, P = 0.047), eGFR (0.95; 0.92–0.99, P = 0.016) and serum sodium (0.70; 0.50–0.91, P = 0.014) were the significant risk factors for decline in LVEF (Table 3). Model performance showed an apparent AUC of 0.88 (95 %CI: 0.81–0.94), while the 10-fold cross-validation and bootstrap validation yielded AUCs of 0.84 (0.75–0.91) and 0.85 (0.78–0.88), respectively.

**Table 3:** Risk factors of decline in LVEF in HFimpEF.

	**Univariable analysis**			**Multivariable analysis**		
	**OR**	**95 % CI**	**p-value**	**aOR**	**95 % CI**	**p-value**
Age	1.01	0.98–1.05	0.519			
Female sex	0.35	0.08–1.10	0.107			
Heart Rate	1.00	0.96–1.03	0.859			
Systolic BP	0.97	0.94–1.00	0.038			
Diastolic BP	0.98	0.94–1.02	0.423			
NYHA class Ⅲ-Ⅳ	4.48	1.24–15.03	0.016			
Ischemic heart disease	1.73	0.69–4.31	0.237			
Atrial fibrillation	1.31	0.52–3.25	0.557			
Cancer	1.21	0.26–4.09	0.774			
Anemia	0.77	0.26–2.00	0.604			
Diabetes mellitus	0.84	0.33–2.06	0.699			
LVEF	0.85	0.78–0.92	<0.001	0.80	0.69–0.90	<0.001
LVDd	1.11	1.05–1.19	<0.001	1.14	1.05–1.25	0.003
IVSTD	0.84	0.67–1.04	0.123			
PWD	0.86	0.68–1.09	0.231			
LAD	1.03	0.98–1.08	0.263			
LVM	1.00	1.00–1.01	0.082			
BNP (per 10 pg/mL)	1.03	1.00–1.05	0.028	1.04	1.00–1.08	0.047
eGFR	0.98	0.96–1.01	0.140	0.95	0.92–0.99	0.016
Serum sodium	0.87	0.73–1.03	0.110	0.70	0.50–0.91	0.014
Serum potassium	1.40	0.53–3.66	0.497			

Abbreviations: 95%CI, 95% confidence interval; aOR, adjusted odds ratio; BNP, B-type natriuretic peptide; BP, blood pressure; eGFR, estimated glomerular filtration rate; IVSTD, interventricular septal thickness at end-diastole; LAD, left atrial diameter; LVDd, left ventricular diastolic dimension; LVEF, left ventricular ejection fraction; LVM, left ventricular mass; NYHA, New York Heart Association; PWD, posterior wall diameter.

## 4 Discussion

We examined the clinical characteristics, prognosis and risk factors regarding decline in LVEF in patients with HFimpEF in our CHART-2 study. The novel findings of our study are as follows; 1) Among the patients with HFrEF, 23 % improved their LVEF (HFimpEF). However, in those with HFimpEF, 12 % had subsequent decline in LVEF associated with a higher incidence of HF hospitalization, 2) The incidence of composite outcomes in the temporary HFimpEF group showed a similar trend to those with the persistent HFrEF group, and 3) LVEF, LVDd, BNP, eGFR and serum sodium were the risk factors for decline in LVEF in patients with HFimpEF. These findings underscore the prognostic significance of monitoring LVEF in patients with HFimpEF.

### 4.1 Clinical characteristics and prognosis of HFimpEF

In the present study, the temporary HFimpEF group was characterized by intermediate characteristics between the persistent HFimpEF and the persistent HFrEF groups. Similar to the previous studies,[18], [19], [20] the HFimpEF group had a higher proportion of women and patients with non-ischemic heart disease. Of note, we found that the patients who were able to preserve LVEF were more likely to be women and had non-ischemic heart disease in those with HFimpEF. Although HFimpEF patients experienced reverse remodeling in cardiac structures during the improvement of LVEF, LV diameters at end-diastole and end-systole in the temporary HFimpEF group were larger than in the persistent HFimpEF group. Furthermore, cardiac reverse remodeling was observed across all the 3 groups from baseline to 1-year follow-up, with the most pronounced remodeling occurring in the persistent HFimpEF group. This finding may help explain why the persistent HFimpEF group had better outcome, suggesting that the degree of reverse remodeling could serve as a prognostic indicator, particularly in patients with HFimpEF.

Of note, the prognosis of the temporary HFimpEF group was similar to that of the persistent HFrEF group. This study showed that not only LVEF improvement but also absolute LVEF values at different time points may have prognostic value, underscoring the importance of longitudinal LVEF assessment in HFimpEF. Consistent with these findings, beta-blocker use at 1-year follow-up was lower in the persistent HFimpEF group. While beta-blockers are a cornerstone therapy for HFrEF,[3] this may reflect the group's distinct clinical characteristics. Compared to the temporary HFimpEF group, they had less ischemic heart disease, atrial fibrillation, and NYHA class III/IV symptoms, along with higher LVEF values, indicating less advanced disease and lower sympathetic drive. Fewer indications for rate control and a more cautious treatment approach in mildly symptomatic patients may have contributed to the lower prescription rate. Thus, the lower beta-blocker use likely reflects their more favorable clinical status rather than a determinant of outcome.

### 4.2 Risk factors for decline in LVEF in HFimpEF

In this study, we identified several risk factors associated with subsequent LVEF decline in patients with HFimpEF, including lower LVEF and larger LVDd, elevated BNP, reduced eGFR, and lower serum sodium at 1-year follow-up. These variables reflect key aspects of cardiac structure, neurohormonal activation, and systemic organ function, all of which contribute to heart failure pathophysiology [6], [21], [22]. Elevated BNP levels indicate persistent myocardial stress and neurohormonal dysregulation despite apparent LVEF improvement, suggesting ongoing cardiac vulnerability that predisposes to functional deterioration. Similarly, a larger LVDd may reflect incomplete reverse remodeling and residual adverse myocardial remodeling, limiting sustained improvement. A relatively lower LVEF at the time of initial improvement may signify limited myocardial reserve and a higher susceptibility to relapse. Additionally, renal impairment and hyponatremia are well-established markers of systemic congestion and neurohormonal imbalance, further compounding cardiac dysfunction [23], [24]. Notably, while previous studies have identified sex and NYHA functional class as predictors of LVEF improvement,[25], [26] our focus on factors associated with decline after improvement revealed no significant associations with these variables, underscoring the distinct mechanisms governing improvement versus relapse in HFimpEF. These findings highlight the complex and multifactorial nature of HFimpEF progression, reinforcing the importance of integrated clinical and biomarker assessment for risk stratification. Future research should clarify molecular mechanisms underlying LVEF decline and evaluate intensified follow-up and tailored therapies to sustain improvement in HFimpEF patients.

### 4.3 Study limitations

The present study has several limitations. First, the relatively small number of patients in the temporary HFimpEF group limits the statistical power to fully characterize the clinical course of this subgroup. In addition, in the exploratory analysis of risk factors for decline in LVEF, five variables were selected as the multivariable model. Given the limited number of events, we performed internal validation with 10-fold cross-validation and bootstrap resampling to assess model stability. The cross-validated and bootstrap-validated AUCs, showed only modest attenuation, suggesting that the model retained reasonable discriminatory ability despite the limited sample size. Therefore, while the findings should still be interpreted with caution, the internal validation procedures support the robustness of the observed associations. Validation in larger and more diverse cohorts is necessary to confirm and extend these observations. Second, the exclusion of patients with missing echocardiographic data from study population may have caused selection bias. As a result, the study population was limited to patients who survived from baseline to the 2-year follow-up, which may represent a relatively healthier subset of patients with HFrEF and could lead to an overestimation of overall prognosis. Third, the lack of central adjudication for LVEF may introduce potential bias and variability in the results. To minimize variability, LVEF measurements in this study were performed according to the guidelines of the European Society of Echocardiography at each participating center [15]. Although variability cannot be completely eliminated, the stratification of outcomes across the three groups, as showed in Supplemental Fig. 1, supports the overall robustness of our findings. Finally, although our study targeted patients with chronic heart failure in a stable outpatient setting, and efforts were made to exclude acute or secondary cardiomyopathies, the presence of unrecognized reversible etiologies cannot be entirely ruled out. In addition, information on drug compliance was not available in the present study. This may have led to the inclusion of patients whose improvement in LVEF reflected not only true reverse remodeling but also improvement from an acute insult or differences in compliance to medical therapy, potentially influencing the interpretation of our findings.

## 5 Conclusions

The present study demonstrates that patients with HFimpEF account for 23 % of those with HFrEF and that 12 % of them have subsequent decline in LVEF associated with similar worse prognosis as in those with persistent HFrEF, highlighting the importance of monitoring LVEF in patients with HFimpEF.

## 6 Disclosures

This work was supported by Health Labour Sciences Research Grant 23FC1050 (Dr. Yasuda) and the Japan Society for the Promotion of Science (JSPS) KAKENHI Grant 22 K11940 (Dr. Miyata).

## 7 IRB Information

The study was approved by the Ethics Committee of Tohoku University Graduate School of Medicine (Reference no. 2023-1-625).

## CRediT authorship contribution statement

**Takuya Takigahira:** Writing – original draft, Visualization, Methodology, Investigation, Formal analysis, Conceptualization. **Kotaro Nochioka:** Writing – review & editing, Writing – original draft, Project administration, Methodology, Investigation, Data curation, Conceptualization. **Satoshi Miyata:** Writing – review & editing, Resources, Project administration, Formal analysis, Data curation. **Takashi Shiroto:** Writing – original draft. **Takumi Inoue:** Writing – review & editing. **Kai Susukita:** Writing – review & editing. **Hideka Hayashi:** Writing – review & editing, Data curation. **Hiroyuki Takahama:** Writing – review & editing. **Jun Takahashi:** Writing – review & editing, Investigation, Data curation. **Hiroaki Shimokawa:** Writing – review & editing, Supervision, Resources, Funding acquisition. **Satoshi Yasuda:** Writing – review & editing, Supervision, Resources, Project administration, Funding acquisition, Conceptualization.

## Declaration of competing interest

The authors declare that they have no known competing financial interests or personal relationships that could have appeared to influence the work reported in this paper.
