# Supplementary material for: Prognostic significance of subsequent decline in LVEF in heart failure with improved ejection fraction − A report from the CHART-2 study −
Source: Int J Cardiol Heart Vasc. 2026 Jan 23;62:101877. doi: 10.1016/j.ijcha.2026.101877 (PMC12861058; doi:10.1016/j.ijcha.2026.101877)
Supplement: Supplementary Data 1 [file mmc1.docx]

**Supplemental Materials**

**Prognostic significance of subsequent decline in LVEF**

**in heart failure with improved ejection fraction**

**- A report from the CHART-2 Study -**

**Supplemental Figure1. Longitudinal changes in LVEF across the three groups**

**
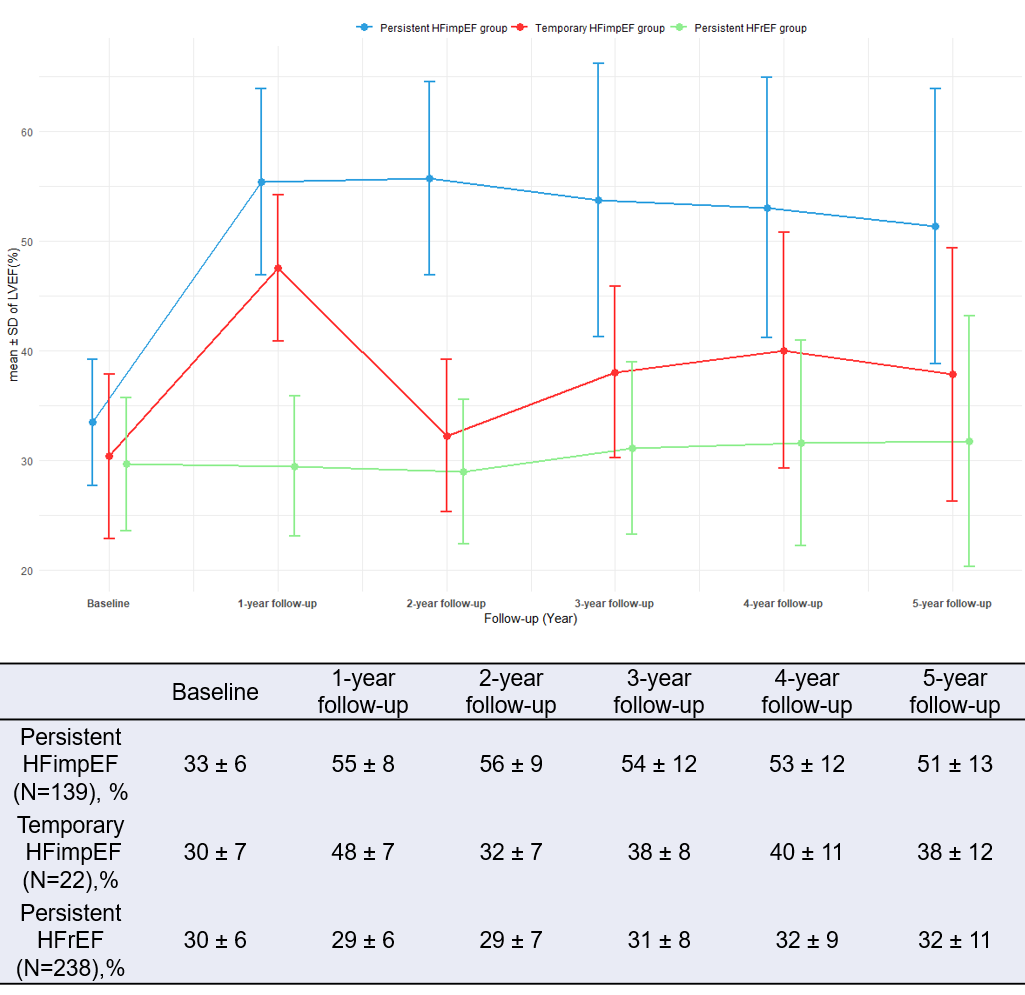
**

**Figure Legends**

Longitudinal changes in LVEF over time among the three groups from baseline to the 5-year follow-up are shown. LVEF values are presented as mean with standard deviation.

Abbreviations: HFimpEF, heart failure with improved ejection fraction; HFrEF, heart failure with reduced ejection fraction; LVEF, left ventricular ejection fraction

**Supplemental Table1. Proportion of medications use at each time point**

|  | Persistent  HFimpEF group (N = 139) | Temporary  HFimpEF group  (N = 22) | Persistent  HFrEF group  (N = 238) |
| --- | --- | --- | --- |
| **Beta-Blockers** |  |  |  |
| Baseline | 88 (63.3%) | 16 (72.7%) | 184 (77.3%) |
| 1-year follow-up | 92 (66.2%) | 18 (81.8%) | 189 (79.4%) |
| 2-year follow-up | 93 (66.9%) | 14 (63.6%) | 191 (80.3%) |
|  |  |  |  |
| **RAS inhibitors** |  |  |  |
| Baseline | 114 (82.0%) | 18 (81.8%) | 206 (86.6%) |
| 1-year follow-up | 107 (77.0%) | 21 (95.5%) | 202 (84.9%) |
| 2-year follow-up | 112 (80.6%) | 19 (86.4%) | 199 (83.6%) |

Abbreviations: HFimpEF, heart failure with improved ejection fraction; HFrEF, heart failure with reduced ejection fraction; RAS, renin-angiotensin system

**Supplemental Table2. Proportion of patients with valvular heart disease who underwent valve surgery by the 1-year follow-up**

|  | Persistent  HFimpEF group  (N = 37) | Temporary  HFimpEF group (N = 4) | Persistent  HFrEF group  (N = 44) | p-value |
| --- | --- | --- | --- | --- |
| Valvular surgery | 7 (18.9%) | 1 (25.0%) | 11 (25.0%) | 0.827 |

Abbreviations: HFimpEF, heart failure with improved ejection fraction; HFrEF, heart failure with reduced ejection fraction

**Supplemental Table3. Proportion of patients categorized the subtypes of cardiomyopathy**

|  | Persistent  HFimpEF group  (N = 55) | Temporary  HFimpEF group  (N = 9) | Persistent  HFrEF group (N = 107) | p-value |
| --- | --- | --- | --- | --- |
| Subtypes |  |  |  | 0.346 |
| DCM | 43 (78%) | 8 (89%) | 95 (89%) |  |
| HOCM | 2 (3.6%) | 0 (0%) | 1 (0.9%) |  |
| Other | 10 (18%) | 1 (11%) | 11 (10%) |  |

Abbreviations: DCM, dilated cardiomyopathy; HCM, hypertrophic cardiomyopathy; HFimpEF, heart failure with improved ejection fraction; HFrEF, heart failure with reduced ejection fraction
